# Application and efficacy evaluation of thoracoscopic techniques in ventricular tumors resection

**DOI:** 10.3389/fcvm.2026.1765033

**Published:** 2026-04-24

**Authors:** Huai Lan, Sijun Cheng, Lihua Lv, Lin Xia, Wenju Yang, Yang Zhao, Zhenlong Wang

**Affiliations:** Department of Cardiovascular Surgery, General Hospital of Northern Theater Command, Shenyang, China

**Keywords:** cardiac tumor resection, fully thoracoscopic surgery, minimally invasive cardiac surgery, thoracoscopy, ventricular tumor

## Abstract

**Background:**

Although fully thoracoscopic resection of atrial tumors has become well established, evidence supporting non-robotic fully thoracoscopic ventricular tumor resection remains limited, largely owing to the deep anatomical location of ventricular lesions, restricted operative space, and the complexity of subvalvular structures.

**Methods:**

We conducted a retrospective analysis of eight patients who underwent right-sided three-port fully thoracoscopic ventricular tumor resection between August 2022 and July 2025. Perioperative outcomes and near-term follow-up results were systematically evaluated.

**Results:**

All patients successfully underwent thoracoscopic tumor resection; one patient concurrently received tricuspid valve repair involving papillary muscle reattachment. Intraoperative transoesophageal echocardiography confirmed complete tumor excision in all cases. No major postoperative complications, including mortality or embolic events, were observed. The cardiopulmonary bypass time was (107 ± 64) min, and the aortic cross-clamp time was (49 ± 39) min. The mean intensive care unit stay was (21.5 ± 4.1) h, mechanical ventilation time was (16.4 ± 2.5) h, and postoperative hospital stay was (5 ± 1) days. No patient required postoperative allogeneic blood transfusion. Echocardiography before discharge and during follow-up demonstrated no residual tumors or significant valvular regurgitation. The mean follow-up duration was (21 ± 12) months, during which no tumor recurrence or distant metastasis was detected.

**Conclusion:**

A right-sided three-port fully thoracoscopic approach allows safe and controlled complete resection of ventricular tumors, with favourable perioperative recovery and near-to-mid-term outcomes. These findings suggest that this technique may represent a promising standardised minimally invasive alternative to conventional open surgery.

## Introduction

Cardiac tumors are rare, with primary cardiac tumors estimated to occur in approximately 0.02% of the population. Among these, benign tumors account for about 75% (1). Common benign cardiac tumors include myxomas, Papillary fibroelastomas, rhabdomyomas, hemangiomas, fibromas, and lipomas (2). Myxomas are the most common primary benign cardiac tumor, comprising >50% of diagnosed cases, and approximately 75% arise in the left atrium. Ventricular myxomas are uncommon, with ventricular involvement reported in only 3% – 4% of myxoma cases (3). Papillary fibroelastomas predominantly involve valvular tissue, with an overall incidence <0.1%, yet they are the second most common benign cardiac tumor in adults. They most often originate from the endocardial surface of the valves, most frequently the aortic valve (approximately 35% – 63%) and mitral valve (approximately 9% – 55%) (4). Papillary fibroelastomas account for approximately 75% of all cardiac valve tumors (5). Hemangiomas are much less common than myxomas and constitute 2.8% of all cardiac tumors (6). Cardiac fibromas are the second most common primary cardiac tumor in children but also occur in adults. Secondary cardiac tumors are more common than primary benign tumors in the general population, with a reported prevalence of approximately 0.1% (7).

In recent years, continued advances in totally thoracoscopic techniques have enabled their application in atrial tumor resection (8), with favourable safety and efficacy outcomes reported (9). In contrast, ventricular tumors pose substantially greater surgical challenges owing to their deeper anatomical location, restricted operative workspace, complex myocardial trabecular architecture, and frequent proximity to critical subvalvular structures, including papillary muscles and chordae tendineae. These factors increase the risk of intraoperative tumor fragmentation and embolisation, thereby requiring a higher level of precision in endoscopic manipulation and significantly increasing procedural complexity. Consequently, minimally invasive treatment of ventricular tumors has largely been limited to isolated case reports or small series describing robot-assisted resections in the existing literature (10). More recently, a limited number of studies have begun to describe fully thoracoscopic ventricular tumor resections performed without robotic assistance, suggesting that ongoing improvements in thoracoscopic visualisation systems and surgical techniques have made intraventricular procedures technically feasible using a totally thoracoscopic approach, with generally acceptable short-term clinical outcomes. For example, Xu et al. reported a series of patients undergoing fully thoracoscopic left ventricular tumor resection (11), while Li et al. described successful resection of a left ventricular myxoma using a similar approach (12). These reports support the technical feasibility of fully thoracoscopic ventricular tumor resection. In addition, several recent small-sample studies have documented favourable early outcomes following fully thoracoscopic resection of benign cardiac tumors (13). Nevertheless, the existing evidence is limited by small cohort sizes, heterogeneity in surgical techniques, and inconsistencies in perioperative and follow-up data reporting, precluding robust or systematic conclusions. At present, high-quality evidence regarding the feasibility, safety, and short- to medium-term outcomes of non-robotic fully thoracoscopic ventricular tumor resection remains insufficient. Thus, although thoracoscopic surgery for atrial tumors has become well established, fully thoracoscopic ventricular tumor resection—particularly in the absence of robotic assistance—remains an evolving and incompletely defined field. Further targeted clinical investigations are therefore required to clarify its feasibility and safety and to systematically evaluate perioperative and short- to medium-term therapeutic outcomes. Accordingly, we conducted a retrospective analysis of perioperative data and near- to mid-term follow-up outcomes in eight patients who underwent ventricular tumor resection using a right-sided three-port fully thoracoscopic approach at our centre, aiming to assess the feasibility and safety of this technique in ventricular tumor management.

## Methods

### Patient selection

This study was approved by the Ethics Committee of the Northern Theater General Hospital [Y (2025) 453]. We enrolled eight patients with ventricular tumors [three right ventricular (RV) and five left ventricular (LV)] who underwent thoracoscopic surgery between August 2022 and July 2025; follow-up was completed in October 2025.

### Diagnosis

**Figure 1:**
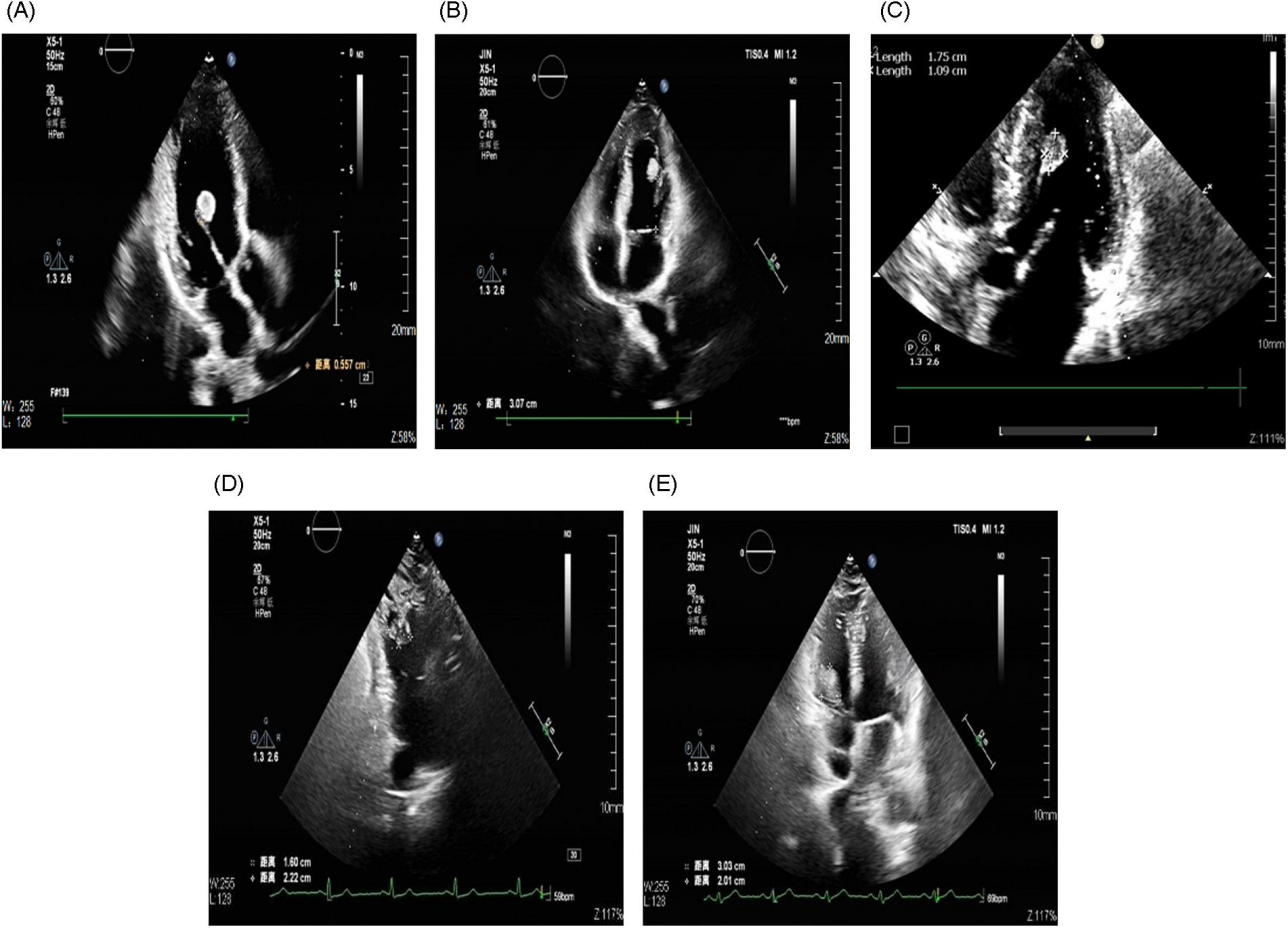
Preoperative TTE findings. **(A)** LV myxoma; **(B)** LV hemangioma; **(C)** LV papillary fibroelastoma; **(D)** RV fibroma; **(E)** RV myxoma.

Preoperatively, tumor size, location, and attachment characteristics were assessed primarily by transthoracic echocardiography (TTE) performed in our institution, with concurrent evaluation of valvular structure and function (14, 15) (Figure 1). Contrast-enhanced cardiac computed tomography (CT) was subsequently performed to delineate anatomic relationships and to help differentiate solid tumors from non-neoplastic intracardiac masses (e.g., thrombus, vegetations, or calcifications) (15). In addition, cross-sectional imaging of the head, chest, and abdomen was performed to assess extra-cardiac organ involvement. In particular, a preoperative head CT scan was routinely performed to evaluate the intracranial condition and to screen for evidence of cerebrovascular embolic lesions. Definitive diagnosis and tumor classification were established by histopathological examination of the resected specimen, which remains the gold standard (16).

### Inclusion criteria

(1) Systolic pulmonary artery pressure (sPAP) on echocardiography is ≤ 60 mmHg; (2) no history of chronic pulmonary disease or prior right thoracic surgery; (3) no concomitant coronary artery, aortic, or aortic valve procedure is required; (4) preoperative chest CT shows no diaphragmatic hernia and no transverse cardiac position; (5) no peripheral vascular disease that would preclude femoral arterial/venous cannulation.

### Exclusion criteria

Patients who did not meet all inclusion criteria were excluded.

### Surgical techniques

**Figure 2:**
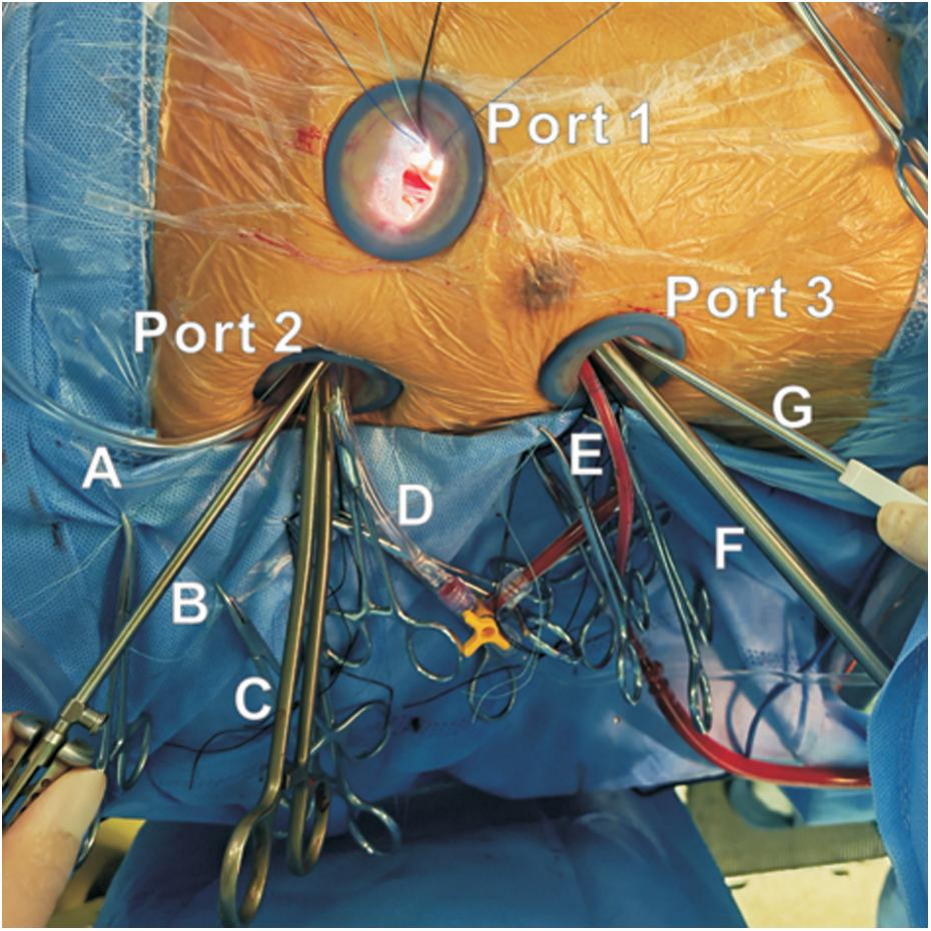
Three-port right-sided thoracoscopic approach: operative ports and instruments. **(A)** CO₂ insufflation line; **(B)** tissue forceps; **(C)** aortic cross-clamp; **(D)** cardioplegia needle; **(E)** left atrial vent; **(F)** thoracoscope; **(G)** intracardiac suction catheter.

General anesthesia was induced with double-lumen endotracheal intubation. The patient was placed in the supine position with a pillow under the back to elevate the right hemithorax by approximately 20°. The right upper limb was positioned parallel to the torso with the elbow slightly flexed to optimize exposure of the right chest wall. After systemic heparinization, peripheral cardiopulmonary bypass (CPB) was established via right femoral arterial and venous cannulation. Three ports were created in the right chest wall: Port 1 (3 cm) was placed in the third intercostal space approximately 2 cm lateral to the right parasternal line to introduce surgical instruments, including tissue scissors and an intracardiac suction catheter. Port 2 (3 cm) was placed in the third intercostal space along the right anterior axillary line for introduction of instruments, including the aortic cross-clamp, tissue forceps, cardioplegia needle, and carbon dioxide (CO₂) insufflation line; Port 3 (3 cm) was placed in the fifth intercostal space lateral to the right midclavicular line for the thoracoscope, left atrial vent, and intracardiac suction catheter (Figure 2). Each port was fitted with an incision protector to maintain patency, facilitate thoracoscopic and instrument manipulation, and minimize injury to the ribs and surrounding tissues.

**Figure 3:**
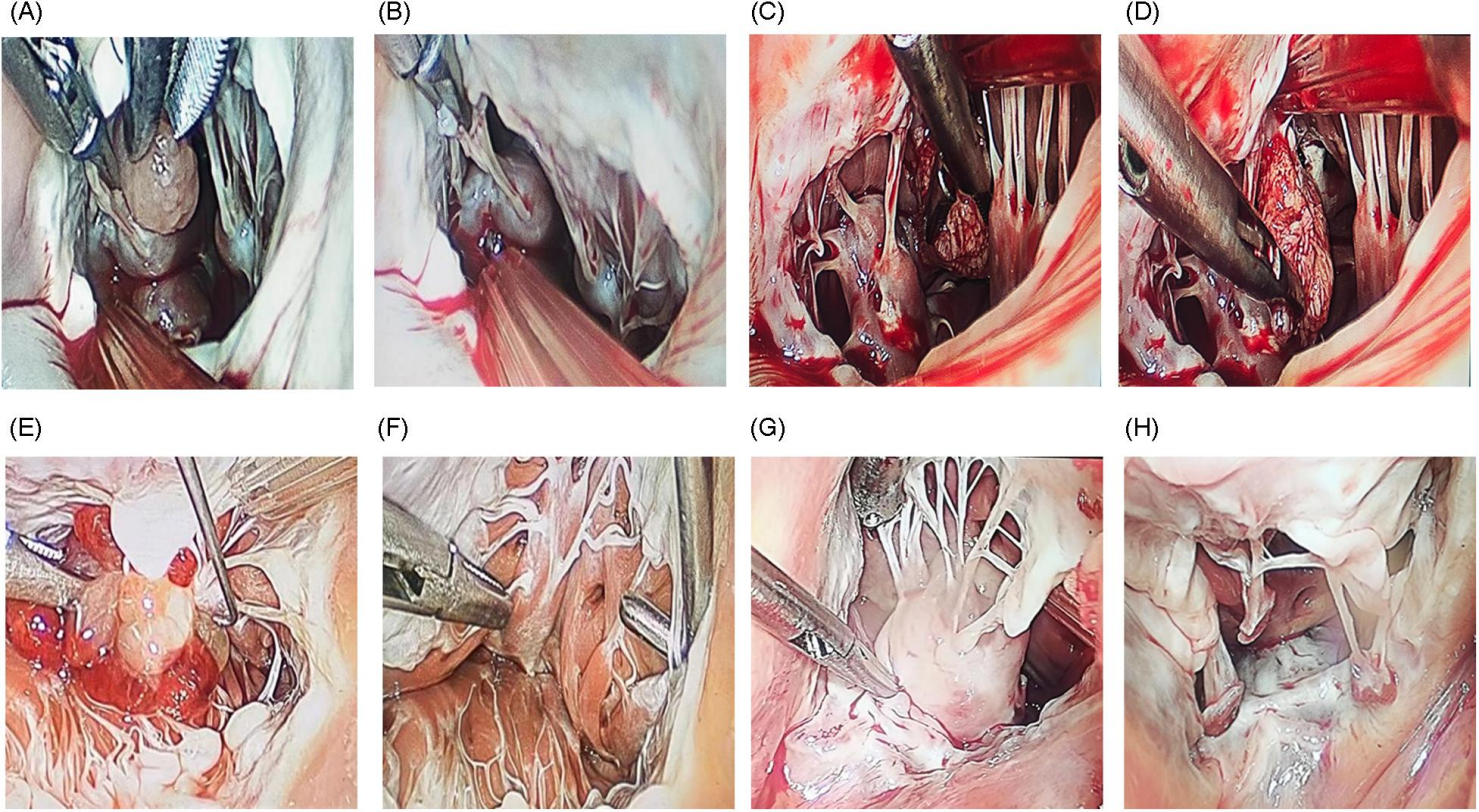
Thoracoscopic ventricular tumor resection. **(A,B)** LV myxoma; **(C,D)** LV papillary fibroelastoma; **(E,F)** RV myxoma; **(G,H)** RV fibroma.

The pericardium was opened approximately 2 cm anterior to the phrenic nerve, and the heart was suspended to optimize exposure. Caval snares were placed around the superior and inferior venae cavae and tightened to achieve bicaval occlusion. A cardioplegia needle was inserted into the aortic root, the ascending aorta was clamped with a Chitwood clamp, and cardioplegia was administered to induce cardiac arrest. Systemic temperature was maintained at 32 °C. A left atrial vent was advanced through the right superior pulmonary vein to improve visualization. Continuous CO₂ insufflation was maintained at 2 L/min via the CO₂ insufflation line. After arrest, the right atrium was opened, and four 4–0 polypropylene traction sutures were placed to retract the atriotomy, providing wide exposure of the tricuspid valve and right atrium. RV tumors were approached through the tricuspid valve. LV tumors were approached via a transseptal route, accessing the LV through the mitral valve; the ventricular cavity was exposed by retracting the mitral or tricuspid leaflets as appropriate. After the tumor and pedicle attachment were identified, the mass was excised completely at its base (Figure 3). Ventricular filling tests were then performed to assess mitral or tricuspid valve competence. If moderate-to-severe regurgitation was present or chordal/leaflet injury was suspected, concomitant valve repair was performed. All cardiac incisions were closed. After de-airing, the aortic cross-clamp was released, and the patient was weaned from CPB. Transesophageal echocardiography (TEE) was performed before separation from CPB to confirm complete resection and adequate mitral and tricuspid valve function. The right femoral arterial and venous cannulas were removed, and protamine sulfate was administered intravenously to reverse heparin. After hemostasis was achieved, a 28-F chest tube was placed through Port 3, and all incisions were closed.

### Statistical analysis

Statistical analyses were performed using SPSS Statistics (version 29.0). Continuous variables are presented as mean ± standard deviation (SD).

## Results

### General clinical features

Eight patients underwent thoracoscopic ventricular tumors resection, including five men and three women, with a mean age of 48 ± 20 years. Preoperative general characteristics were as follows: mean height 169 ± 5 cm, weight 71 ± 6 kg, body mass index (BMI) 25.3 ± 3.0 kg/m^2^, cardiothoracic ratio (CTR) 0.51 ± 0.04, ejection fraction (EF) 60 ± 1%, and sPAP 34 ± 3 mmHg. Preoperative lower-extremity vascular ultrasonography showed mean right femoral artery and vein diameters of 7.7 ± 0.9 mm and 8.1 ± 1.0 mm, respectively. New York Heart Association (NYHA) functional class was I in four patients, II in three, and III in one (Table 1).

**Table 1:** Baseline characteristics of patients with ventricular tumors.

Variable	RV Fibroma	RV Myxoma	RV Myxoma	LV Hemangioma	LV Myxoma	LV Myxoma	LV Myxoma	LV Papillary fibroelastoma	Mean ± SD
Gender	Male	Male	Female	Male	Female	Female	Male	Male	NA
**Age, y**	69	64	71	29	16	40	45	53	48 ± 20
**Height, cm**	168	170	162	179	168	165	170	168	169 ± 5
**Weight, kg**	78	70	82	74	67	72	64	64	71 ± 6
**BMI, kg/m2**	27.6	24.2	31.2	23.1	23.7	26.4	22.1	23.8	25.3 ± 3.0
**CTR**	0.55	0.46	0.52	0.46	0.45	0.52	0.54	0.56	0.51 ± 0.04
**Maximum tumor diameter, mm**	22	30	54	18	30	39	16	21	29 ± 13
**EF, %**	61	60	62	60	61	60	60	59	60 ± 1
**sPAP, mmHg**	36	38	34	34	32	35	32	27	34 ± 3
**Right femoral artery diameter, mm**	7.2	8.2	8.6	8.8	7.5	7.6	8	5.9	7.7 ± 0.9
**Right femoral vein diameter, mm**	8	7.5	8.6	9.8	8.4	8.7	7.6	6.4	8.1 ± 1.0

RV, right ventricle; LV, left ventricle; BMI, body mass index; CTR, cardiothoracic ratio; sPAP, systolic pulmonary artery pressure; EF, ejection fraction.

### Tumor size and location

**Figure 4:**
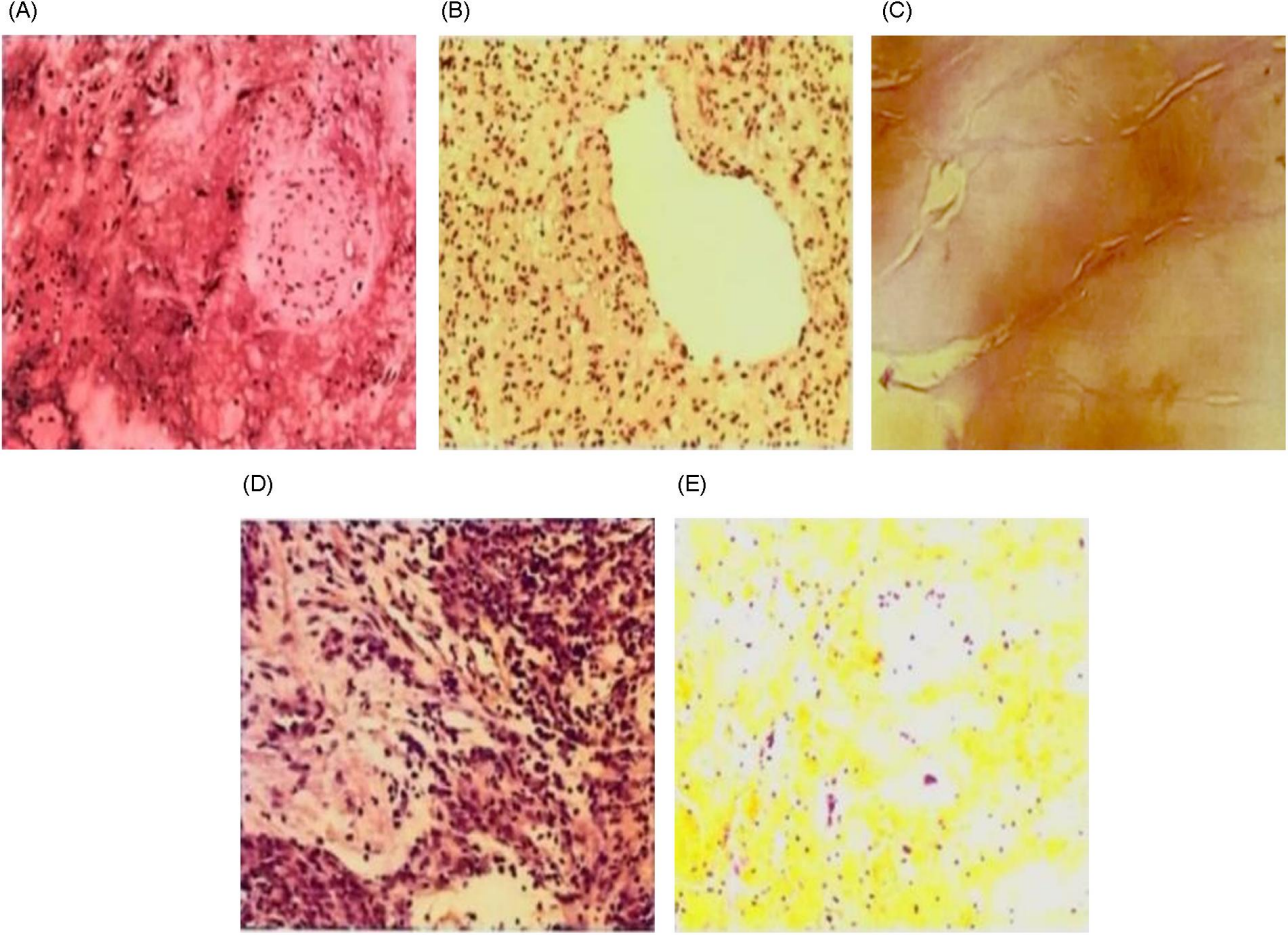
Pathological images of ventricular tumors. **(A)** LV myxoma; **(B)** LV hemangioma; **(C)** LV papillary fibroelastoma; **(D)** RV fibroma; **(E)** RV myxoma.

The mean maximum tumor diameter was 29 ± 13 mm. Three tumors were located in the RV: (1) RV fibroma, with the pedicle attached to the base of the anterior papillary muscle and tightly intertwined with the papillary muscle; (2) RV myxoma, with the pedicle located at the apex of the posterior papillary muscle; and (3) RV myxoma, with the pedicle attached to the anteroseptal commissure of the tricuspid valve with focal adhesions at the attachment site. Five tumors were located in the LV: (1) LV hemangioma, with the pedicle attached to the anterior LV wall approximately 30 mm from the mitral annulus; (2) LV myxoma, with the pedicle at the junction of the inferior and anterior walls approximately 16 mm from the apex; (3) two LV myxomas, with pedicles at the base of the anterolateral papillary muscle; and (4) LV papillary fibroelastoma, with the pedicle located in the mid interventricular septum. This study includes representative histopathological images to illustrate the characteristic findings across pathological subtypes (Figure 4).

### Perioperative outcomes

**Figure 5:**
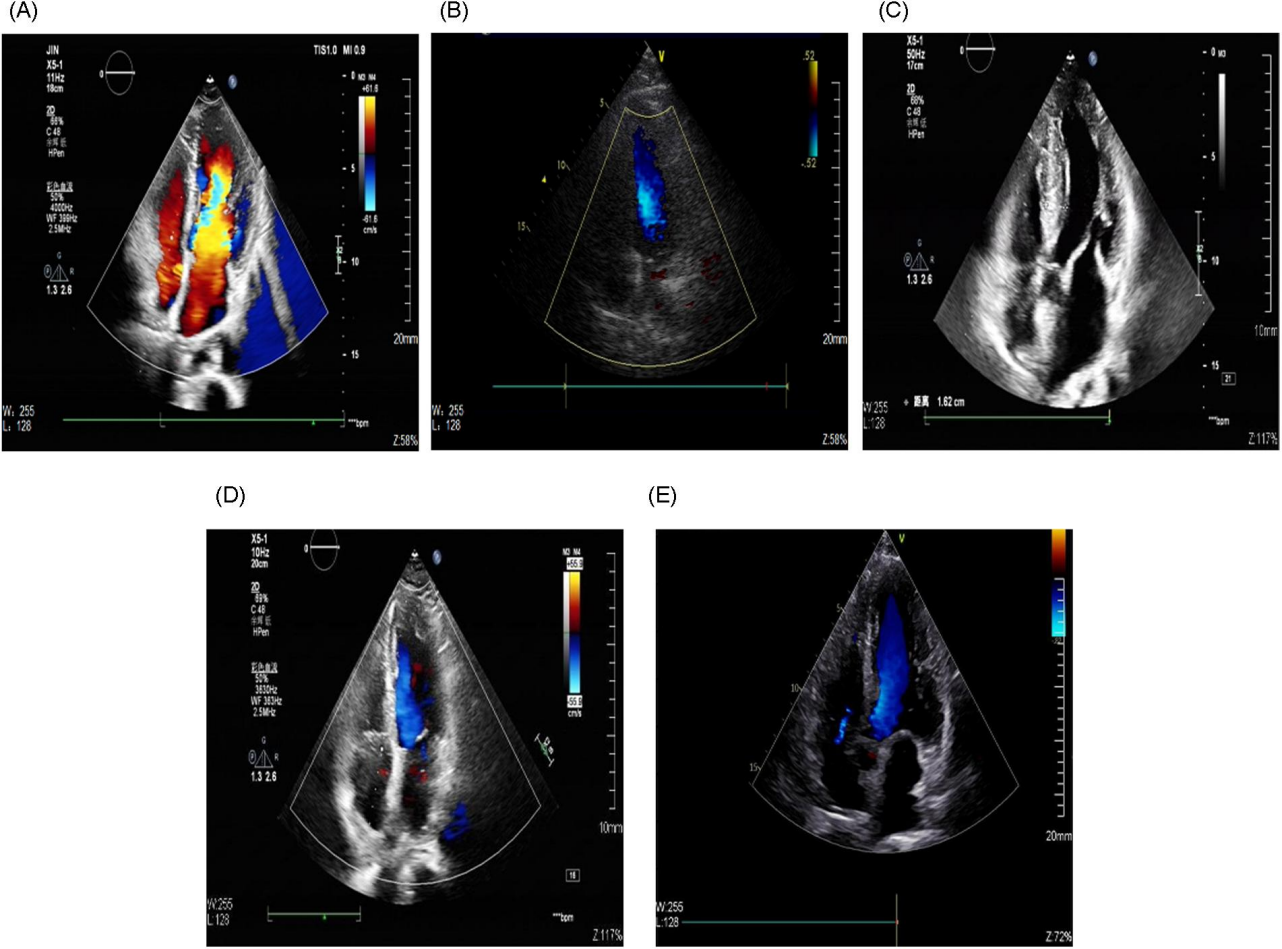
Pre-discharge TTE findings. **(A)** LV myxoma; **(B)** LV hemangioma; **(C)** LV papillary fibroelastoma; **(D)** RV fibroma; **(E)** RV myxoma.

All patients underwent successful thoracoscopic tumors resection. Intraoperative TEE confirmed complete excision without residual lesions. One patient with a RV fibroma underwent concomitant tricuspid valve repair (papillary muscle reattachment). Mean CPB time was 107 ± 64 min, mean aortic cross-clamp time was 49 ± 39 min, and mean operative time was 3.4 ± 1.4 h. No major perioperative adverse events, including death or embolism, occurred. Mean intensive care unit (ICU) stay was 21.5 ± 4.1 h, mean duration of mechanical ventilation was 16.4 ± 2.5 h, and mean postoperative length of stay was 5 ± 1 days. Mean total postoperative thoracic drainage volume was 241 ± 56 mL (Table 2). Before discharge, TTE showed no residual tumor and no clinically significant valvular regurgitation in any patient (Figure 5). Preoperative head CT was performed in all patients as part of the routine preoperative evaluation. No patient developed postoperative symptomatic neurological deficits.

**Table 2:** Perioperative and follow-up data for patients with ventricular tumors.

Variable	RV Fibroma	RV Myxoma	RV Myxoma	LV Hemangioma	LV Myxoma	LV Myxoma	LV Myxoma	LV Papillary fibroelastoma	Mean ± SD
**CPB time, min**	264	85	97	72	87	96	80	77	107 ± 64
**Aortic cross-clamp time, min**	140	41	25	24	44	58	33	25	49 ± 39
**Operation time, h**	6.7	2.7	3.2	2.9	3.0	3.8	2.7	2.2	3.4 ± 1.4
**ICU stay, h**	23.6	17.3	16.7	27.4	21.1	25.7	17.3	22.7	21.5 ± 4.1
**Mechanical ventilation time, h**	16.8	16.2	15.8	16.8	15.1	12.4	16.5	21.3	16.4 ± 2.5
**Postoperative hospital stay, d**	6	5	4	5	4	5	4	5	5 ± 1
**Drainage, mL**	270	310	140	215	260	300	200	235	241 ± 56
**Follow-up time, mo**	35	24	16	37	22	21	7	3	21 ± 12
**EF at follow-up, %**	60	59	60	59	59	55	58	57	58 ± 2
**sPAP at follow-up, mmHg**	31	30	37	37	28	34	34	21	32 ± 5

RV, right ventricle; LV, left ventricle; CPB, cardiopulmonary bypass; ICU, intensive care unit.

### Follow-up

As of October 2025, the mean follow-up duration was 21 ± 12 months, and no tumor recurrence or distant metastasis was observed. Follow-up TTE showed a mean EF of 58 ± 2%, sPAP of 32 ± 5 mmHg, and no clinically significant valvular regurgitation. NYHA functional class was I in five patients and II in three. Overall, cardiac function recovered well, and no new-onset arrhythmias were documented (Table 2).

## Discussion

Cardiac tumors are rare but can be life-threatening. As disease progresses, some benign tumors may enlarge into the cardiac chambers, leading to outflow obstruction and valvular dysfunction. Partial or complete tumor detachment can precipitate acute embolism or sudden cardiac death (17). Clinical presentations range from asymptomatic disease to obstructive, embolic, and/or systemic manifestations, depending on tumor size, location, and its relationship to adjacent cardiac structures. Myocardial infiltration may present as congestive heart failure (15, 18). Tumors adjacent to the atrioventricular node or conduction system may cause atrioventricular block or other arrhythmias. Thrombi or tumor fragments entering the systemic circulation can result in cerebrovascular events or peripheral arterial embolism, whereas embolization to the pulmonary circulation may cause pulmonary embolism (19). Owing to the greater contractile excursion of the ventricles, ventricular tumors are more prone to fragmentation and embolization than atrial tumors (20). Therefore, once a ventricular tumor is diagnosed, prompt surgical intervention is warranted.

Currently, surgical resection remains the most common and effective treatment for cardiac tumors (21). With advances in medical technology and the adoption of minimally invasive concepts, median sternotomy no longer confers an absolute advantage. Prior studies suggest that, while maintaining high rates of complete resection and favorable long-term survival, thoracoscopic cardiac surgery may reduce perioperative mortality and postoperative pain, shorten hospital stay, and potentially lower overall costs (22, 23). With respect to patient selection, thoracoscopic approaches may be particularly beneficial for higher-risk patients, including older adults and those with obesity (24), with perioperative mortality and recurrence rates comparable to, or lower than, conventional open surgery (25). Robotic surgery has also been used for left and RV myxoma resection with favorable outcomes (26, 27). However, systematic evidence supporting the feasibility and safety of fully thoracoscopic ventricular tumor resection without robotic assistance remains limited. The findings of the present study demonstrate that a right-sided three-port thoracoscopic approach can provide adequate surgical exposure and high-resolution visualisation, thereby enabling safe, precise, and controllable intracardiac manipulation to achieve complete resection of ventricular tumors.

Adequate surgical exposure and clear visualisation are fundamental to successful ventricular tumor resection. Compared with atrial tumors, ventricular lesions are more strongly influenced by myocardial contractile motion and are frequently partially obscured by myocardial trabeculae, papillary muscles, or valvular structures, thereby increasing procedural complexity. In conventional median sternotomy, exposure is typically achieved through extensive atrial incisions and manual retraction; however, this advantage is partially offset by increased surgical trauma. In contrast, the fully thoracoscopic approach effectively compensates for the restricted operative workspace by providing high-definition, magnified, and multi-angle visualisation of the surgical field, enabling detailed assessment of the ventricular cavity and its adjacent structures (27). Based on our experience, the enhanced magnification and visual clarity afforded by thoracoscopy facilitate precise identification of tumor morphology, pedicle location, and depth of attachment, which is particularly critical for tumors with a fragile consistency or those located adjacent to papillary muscles. Moreover, dynamic adjustment of camera angles allows near-panoramic visualisation of the ventricular cavity, thereby minimising blind spots and reducing the risk of incomplete resection or inadvertent injury to critical intracardiac structures.

A key contribution of the present study is the systematic description and successful implementation of a right-sided three-port fully thoracoscopic approach for tumor resection, which demonstrated a high degree of reproducibility. This configuration enables stable triangular instrument alignment, facilitating efficient coordination among the thoracoscope, intracardiac suction device, and dissection instruments. Compared with hybrid mini-thoracotomy or port-assisted techniques, the fully thoracoscopic approach minimises thoracic wall trauma while preserving sufficient and well-controlled operative workspace. According to the specific anatomical location of the tumor, right and left ventricular lesions are addressed through either transvalvular or trans–interatrial septal approaches. Notably, the high-definition, magnified thoracoscopic visual field allows precise and controlled manipulation of the valve leaflets, thereby avoiding excessive traction and reducing the risk of chordal or leaflet injury. Intraoperative valve function is systematically evaluated using ventricular filling tests, and concomitant valve repair is performed when functional impairment or a risk of structural damage is identified.

In ventricular tumor surgery, particularly for myxomas and papillary fibroelastomas, tumor fragmentation and the associated risk of embolisation remain major concerns. Owing to stronger ventricular contractility and more complex intracavitary anatomy, these risks may be further amplified during ventricular tumor procedures. Under thoracoscopic guidance, we adopted a tumor-handling strategy that prioritised early pedicle dissection, thereby avoiding direct compression or excessive manipulation of the tumor body. High-definition magnification of the operative field enabled precise dissection at the tumor base, facilitating complete resection while minimising mechanical disturbance. All resected specimens were immediately placed into an endoscopic retrieval bag and extracted through port 1, further reducing the risk of tumor fragmentation and embolic events. Notably, no perioperative embolic complications were observed in this series, supporting the safety of this thoracoscopic tumor management strategy in ventricular tumor resection.

Beyond technical feasibility, thoracoscopic surgery is also associated with favourable perioperative recovery outcomes. In the present study, a right-sided three-port thoracoscopic technique was used to perform ventricular tumor resection in eight patients. The mean intensive care unit (ICU) stay was (21.5 ± 4.1) h, which was substantially shorter than the approximately 48 h reported for patients undergoing median sternotomy in previous studies (28). Similarly, the mean postoperative hospital stay was (5 ± 1) days, representing a significant reduction compared with conventional open surgery (29). Previous evidence indicates that large-volume allogeneic blood transfusion is an independent risk factor for hospital-acquired pneumonia after cardiac surgery and is closely associated with postoperative acute kidney injury (30). Notably, none of the patients in this series required postoperative allogeneic blood transfusion. This finding may reflect the reduced surgical trauma, limited chest wall disruption, and more effective haemostasis achieved under the clear and magnified visualisation provided by thoracoscopy. Collectively, these results suggest that, when performed by an experienced surgical team, fully thoracoscopic ventricular tumor resection may offer meaningful advantages in perioperative recovery.

Robot-assisted technology has been applied to ventricular tumor resection and offers advantages in instrument articulation and precision. However, the widespread adoption of robotic systems remains limited, as their use is typically associated with higher costs as well as complex preoperative preparation and system setup. Based on our experience, non-robotic, fully thoracoscopic surgery can achieve surgical exposure and clinical outcomes comparable to those of robotic-assisted approaches when supported by appropriate port placement and well-established thoracoscopic techniques, without reliance on a robotic platform. Compared with conventional open surgery, thoracoscopic procedures substantially reduce surgical invasiveness while preserving oncological completeness. Accordingly, for patients whose anatomical characteristics and physiological status are suitable for minimally invasive intervention, fully thoracoscopic ventricular tumor resection represents a compelling alternative.

Preliminary findings from the present study suggest that a right-sided three-port thoracoscopic approach can be safely and effectively applied for ventricular tumor resection. By providing excellent surgical exposure, precise and controllable intracardiac manipulation, and favourable perioperative recovery, this technique extends the application of minimally invasive cardiac surgery from atrial tumors to ventricular tumors. With continued progression along the learning curve, ongoing refinement of surgical techniques and instruments, and further validation through multicentre studies, fully thoracoscopic ventricular tumor resection may gradually evolve into a standardised minimally invasive alternative to conventional open-heart surgery.

## Limitations

This study has several limitations. First, as a single-center retrospective case series, it is subject to selection and information bias. In addition, the small sample size and relatively short follow-up preclude robust conclusions regarding long-term recurrence and survival after thoracoscopic resection of ventricular tumors across pathological subtypes. Second, although preoperative head CT was performed, routine postoperative cerebral imaging was not conducted in asymptomatic patients. Therefore, while no patient developed clinically evident neurological deficits, silent cerebral infarction or microembolic events could not be completely excluded. Third, this technique requires substantial expertise in thoracoscopic manipulation and CPB surgery, as well as strong multidisciplinary coordination. Given the steep learning curve, its generalizability and reproducibility across centers require further validation. Multicenter, prospective studies with larger cohorts, longer follow-up, and standardized perioperative neuroimaging assessment are needed to better define the long-term outcomes and clinical benefits of fully thoracoscopic ventricular tumor resection.

## Conclusions

This study demonstrates that the three-port fully thoracoscopic technique for ventricular tumors resection, performed by a team with experience in thoracoscopic cardiac surgery, offers good feasibility and safety. It shortens ICU and hospital stays while ensuring complete tumor resection, and reduces allogeneic blood transfusions and related complications. Mid-term follow-up revealed favorable cardiac function recovery in patients, with no evidence of tumor recurrence or distant metastasis. These findings suggest this technique holds promise as an effective minimally invasive surgical option for ventricular tumor patients.

## Data Availability

The original contributions presented in the study are included in the article/Supplementary Material, further inquiries can be directed to the corresponding author.
